# Structure and Functions of HMGB2 Protein

**DOI:** 10.3390/ijms24098334

**Published:** 2023-05-05

**Authors:** Tatiana Starkova, Alexander Polyanichko, Alexey N. Tomilin, Elena Chikhirzhina

**Affiliations:** Laboratory of Molecular Biology of Stem Cells, Institute of Cytology of the Russian Academy of Sciences, Tikhoretsky Av. 4, 194064 St. Petersburg, Russia

**Keywords:** non-histone protein HMGB2, DNA–protein interactions, protein–protein interactions, structure and functions of HMGB2

## Abstract

High-Mobility Group (HMG) chromosomal proteins are the most numerous nuclear non-histone proteins. HMGB domain proteins are the most abundant and well-studied HMG proteins. They are involved in variety of biological processes. HMGB1 and HMGB2 were the first members of HMGB-family to be discovered and are found in all studied eukaryotes. Despite the high degree of homology, HMGB1 and HMGB2 proteins differ from each other both in structure and functions. In contrast to HMGB2, there is a large pool of works devoted to the HMGB1 protein whose structure–function properties have been described in detail in our previous review in 2020. In this review, we attempted to bring together diverse data about the structure and functions of the HMGB2 protein. The review also describes post-translational modifications of the HMGB2 protein and its role in the development of a number of diseases. Particular attention is paid to its interaction with various targets, including DNA and protein partners. The influence of the level of HMGB2 expression on various processes associated with cell differentiation and aging and its ability to mediate the differentiation of embryonic and adult stem cells are also discussed.

## 1. Introduction

The non-histone protein HMGB2 is one of the HMGB domain proteins, which are a part of the HMG superfamily (High-Mobility Group). HMG proteins were first discovered in mid 1970s as by-products of histone isolation high electrophoretic mobility in a polyacrylamide gel, hence the name [1]. According to their structures and functions, HMG proteins are divided into three families [2,3]: HMGA (formerly known as HMG-I/Y), HMGN (formerly known as HMG-14/17) and HMGB (formerly known as HMG-1/2). HMGA proteins contain an AT-hook motif and interact with AT-rich regions of the minor groove in DNA. The AT-hook motif is evolutionarily conservative from bacteria to humans. HMGA proteins take part in transcription processes, as well as in the regulation of the chromatin structure [4,5]. HMGN proteins are directly associated with nucleosomes and participate in transcription initiation, although they are not part of the transcription complex [6,7].

Members of the HMGB family interact with DNA through highly conserved DNA-binding domains known as HMGB domains. Besides HMG-1/2 themselves and recently discovered homologues, HMGB3 and HMGB4 [8], the HMGB family includes all proteins that contain one or more HMGB domains with a characteristic L-shaped structure in their structure [9,10]. Within the HMGB family, proteins can also be divided into two groups. The first group consists of single-domain HMGB proteins, which are less common in chromatin than those with multiple domains are. Single-domain HMGB proteins include proteins such as TCF/LEF-1, TOX, sex-determining factor SRY, proteins of the SOX subfamily, chromatin modeling factors BAF57 and PB1 [11]. Single-domain HMGB proteins are characterized by sequence-specific DNA binding, which is due to the formation of a small number of non-covalent bonds between single-domain HMGB and nitrogenous bases in the minor groove upon interaction [8,12,13]. Proteins containing two or more HMGB domains, such as HMGB1-4, mitochondrial factors mtTF1, and UBF, a transcription factor of RNA polymerase I, are more common [8,11,12,13]. One of the important characteristics of multi-domain HMGB proteins is the lack of sequence specificity in DNA recognition; instead, these proteins show specificity for the DNA structure [14].

Among all HMGB proteins, two of the most common and well-studied ones are HMGB1 and the closely related HMGB2, in which 80% of amino acid sequences are identical [8,12,13]. Early studies of the functions of the HMGB1 and HMGB2 proteins indicated, first of all, their DNA-binding activity and participation in the structural organization of chromatin [2]. Later, it was demonstrated that HMGB proteins participate in the remodeling of the chromatin structure [8,15]. The development of these works led to a better understanding of the regulatory mechanisms of cellular processes at the chromatin level and the role of HMGB proteins in processes such as transcription [13] and DNA repair [8,16]. Later, it was found that the functions performed by HMGB1 and HMGB2 depend on their cellular localization [17,18,19]. Depending on the redox status of proteins and their post-translational modifications, HMGB1 and HMGB2 proteins can pass into the cytosol with subsequent secretion into the extracellular space. For HMGB1, the involvement of the extracellular protein in signal transduction processes in the case of immune and anti-inflammatory responses was shown [20,21,22,23]. Extracellular HMGB1 acts as a signaling molecule, promotes the release of anti-inflammatory cytokines, and thus, participates in the body’s response to inflammation, sepsis, oncological and autoimmune diseases [20,23,24,25,26,27,28,29], which makes this protein one of the class of alarmins. Alarmins are molecules that are released from cells after trauma, during inflammatory processes and during viral diseases. These signaling molecules play an important role, for example, in enhancing the body’s inflammatory response to the SARS-CoV-2 infection [26]. Because of the severe consequences and high mortality of patients with COVID-19, the early detection of high levels of alarmins is beneficial for disease treatments. As such, high levels of HMGB1 and illexins are signs of a severe lung injury and fibrotic lesions, indicating the need for supplemental oxygen. *HMGB1* knockout has also been shown to protect cells from SARS-CoV-2-induced death, and the degree of this protection correlates with the HMGB1 levels [30]. HMGB1 may be involved in COVID-19 through at least two mechanisms: the TLR4-mediated cytokine storm in immune cells and RAGE-mediated ACE2 expression in alveolar epithelial cells [29]. We were unable to find similar studies on the role of HMGB2; however, due to the high structural homology of HMGB1 and HMGB2 proteins, the possible role of the latter one in the course/development of COVID-19 cannot be completely ruled out.

Initially, after the discovery of the HMGB1 and HMGB2, it was found that these proteins are similar in both amino acid sequence and structure [8,11,12,13]. Historically, researchers’ attentions have focused primarily on the HMGB1. To date, a fairly large amount of data have been gathered regarding the structure and functions of this protein [2,8,11,13,18,20,22]. Much later, after the development of new methods of cell and molecular biology (such as the knockout and knockdown, RNA-seq, RNA interference, etc.), it became clear that the initial idea of the similarity of the functions of HMGB1 and HMGB2 is not entirely correct, and the full picture is much more complicated. Although HMGB2 is still less studied and characterized than HMGB1, the data accumulated to date increasingly point to differences in the expressions and functions of these two structurally similar proteins. The molecular structure of the HMGB1 protein, its post-translational modifications and the mechanisms of its interaction with other proteins and DNA in cells have been described in greater detail elsewhere [11]. In this review, we focus on the structural and functional properties of HMGB2 protein, as well as on its role in cancer and autoimmune diseases.

## 2. Structural Organization of HMGB2

**Figure 1:**
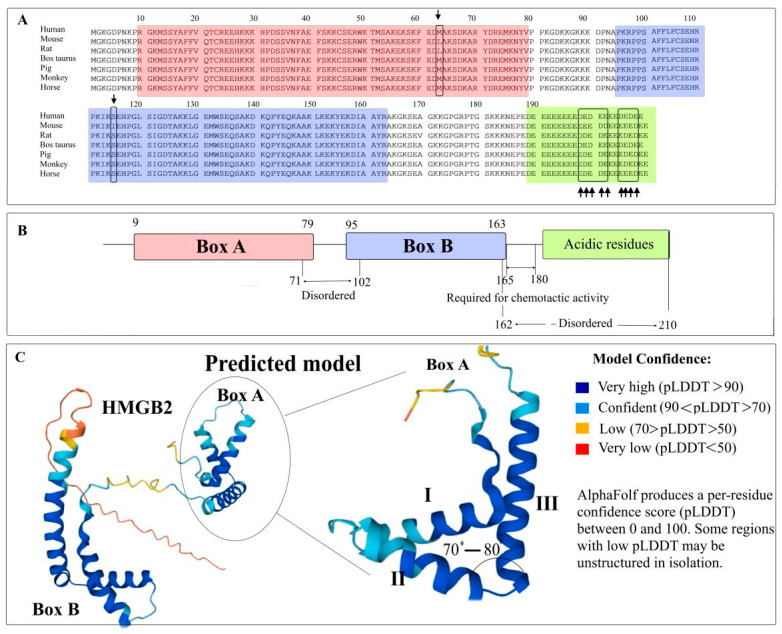
Structure of the mammalian HMGB2 proteins. Panel (**A**)—amino acid sequence alignment of the HMGB2 proteins (the sequences retrieved from UniProt database). The DNA-binding domains (Box A and Box B) are marked with red and blue boxes, respectively. Light green box corresponds to the C-terminal acidic region. The arrows indicate the sites of amino acid substitutions. Panel (**B**)—the schematic representation of HMGB2 domains. Panel (**C**)—tertiary structure of HMGB2 predicted using AlphaFold [33]. I, II and III—three α-helices within the Box A.

Human HMGB2 consists of 209 amino acid residues. The primary structure of the HMGB2 protein is shown in Figure 1, and it is characterized by high interspecific conservatism across mammals: the amino acid sequences of HMGB2 proteins of the studied organisms are almost identical (Figure 1A). The tertiary structure of HMGB2 is represented by a short N-terminal fragment, two DNA-binding HMGB domains, A and B, connected by a small linker and a long disordered C-terminal region (Figure 1B) [31,32]. HMGB domains of the protein (Boxes A and B, 9–79 and 95–163 aa, respectively) are rich in positively charged amino acid residues and carry a total charge of +20 at a neutral pH. The structures of A (PDB ID 1J3X) and B (PDB ID 1J3D) domains of the HMGB2 protein are very similar. There are three α-helical regions in their structure, forming short (31 Å, helices I and II) and long (36 Å, helix III) arms positioned at an angle of ~70–80° relative to each other (Figure 1C). The orientation of the two arms is fixed by strong hydrophobic interactions between amino acid residues located at the corner apex. An additional mechanism of stabilization might involve interaction between proline residues in 5th and 8th positions at the N-terminal region and the inner surface of helix III of A domain. However, according to the NMR structure of the isolated domain, this region demonstrates extreme flexibility [9,10,32].

The C-terminal region (186–209 aa) of the protein includes a continuous sequence of Asp and Glu dicarboxylic amino acid and is negatively charged under physiological conditions. The main differences between HMGB1/2 proteins reside in the length of the C-terminal region and the conservative in D to E substitutions within this region (Figure 1) [34].

According to published data, the C-terminal region of HMGB1 and 2 modulates their interaction with DNA and other proteins [11,13,34,35,36,37,38,39]. The C-terminus of HMGB1 binds to the N-terminal region of H1, disrupting its interactions with DNA and leading to the displacement of H1 [39,40,41], facilitating the sliding of a nucleosome along the DNA, which is essential for the binding of transcription factors [35,42,43,44,45,46,47,48]. It has been shown that tumor suppressor p53 protein interacts with the Box A of HMGB1 [49,50] and that this interaction is regulated by the C-terminal tail [51]. It is interesting to note that HMGB1 was identified as a component antibacterial secrete of human adenoids [52] and, according to Gong et al., its antibacterial activity is associated with the sequence of 201–205 aa within the C-terminal region of the protein [53].

The combination of experimental data and the results of theoretical predictions indicate that the C-terminal region of the protein is located in the cavity between two DNA-binding domains [33,35,36,54,55,56,57].

## 3. Location in the Genome and Expression Level of HMGB2 at Different Stages of Ontogenesis

**Figure 2:**
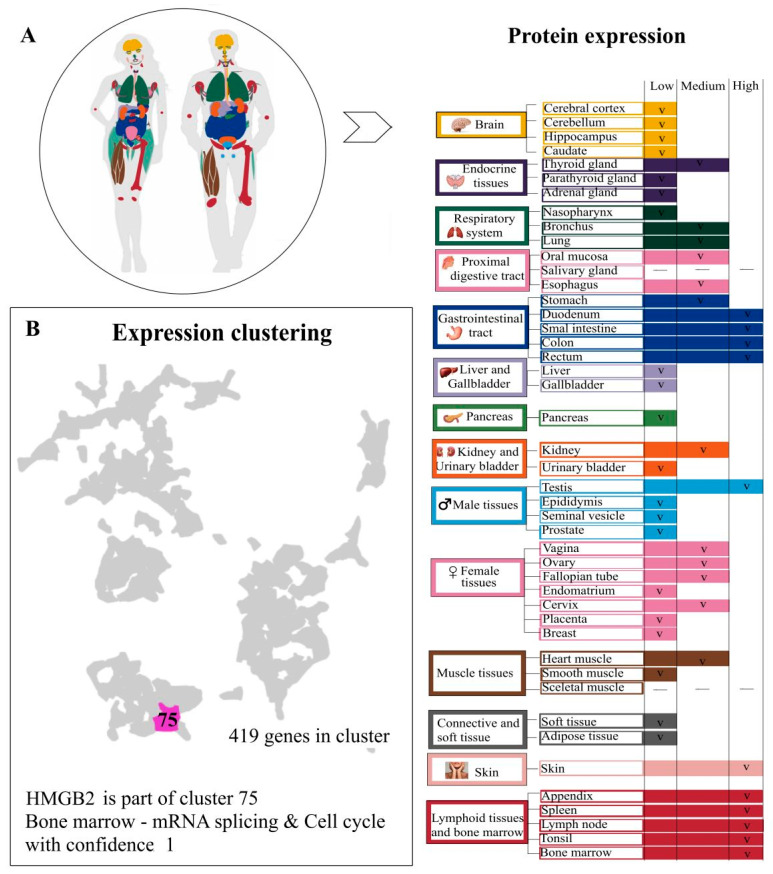
The levels of HMGB2 expression in different tissues and expression clustering. Panel (**A**)—The levels of expression of HMGB2 in an adult organism vary. Skin cells, appendix, spleen, lymph nodes, tonsils, bone marrow, thyroid gland, intestinal tissues and testes are characterized by a high level of protein expression [59,60,61], while cerebral cortex, cerebellum, liver, pancreas, gallbladder, soft and adipose tissues show low levels of HMGB2 [56]. It has been established that HMGB2 is highly expressed in undifferentiated myoblasts and contributes to the restoration of muscle tissue [62]. Interestingly, HMGB2 is also expressed in immortalized human and mouse cells, possibly helping to overcome replicative arrest [63]. Panel (**B**)—RNA expression data were used to classify protein-coding genes into expression clusters for tissues. For dataset, genes detected at nTPM > 1. To account for differences in dynamic ranges between genes across samples, data were gene-wise converted into z-scores. After that, expression data were translated into a lower dimensional space using Principal Component Analysis (PCA). The number of components was selected to satisfy Kaiser’s rule. Gene-to-gene distances were calculated using the Spearman correlation of gene expression across samples and transformed to Spearman distance. The distances were transformed into a shared nearest neighbor graph and used for Louvain clustering to find clusters of genes with similar expression profiles within the graph. Confidence of the gene-to-cluster assignment was calculated as the fraction of times that the gene was assigned to the cluster. A confidence of 1 indicates that the gene was assigned to this cluster in all repeated clusterings. The cluster annotation is based on overrepresentation analysis towards biological databases, including Gene Ontology, Reactome, PanglaoDB, TRRUST and KEGG, as well as HPA. The clustering results are displayed in a UMAP. HMGB2 is part of cluster 75. The 15 nearest neighbors of HMGB2, based on tissue RNA expression, are presented at Table 1. The figure is based of data from the human protein atlas [58].

HMGB2, like its closely related protein, HMGB1, is a DNA-binding protein that is widely distributed in chromatin—on average, one protein molecule per 10–15 nucleosomes [13]. One of the characteristic features of the HMGB2 protein resides in its tissue specificity. In mammals, the HMGB2 (as well as HMGB1 and HMGB3) is highly expressed in all tissues during embryogenesis. The transition from fetus to the adult state is accompanied by the maintenance of or decrease in the expression levels of HMGB1 and HMGB2. In postnatal mammals, the high level of the HMGB2 expression is retained (at different levels) only in a few tissues such as the testicles, ovaries, and lymphoid tissue (Figure 2) [58]. RNA expression data have been used to classify protein-coding genes into expression clusters for tissues. Clustering is splitting a set of objects into non-intersecting subsets-clusters, so that in each of the subsets, the objects are as close as possible to each other according to some criterion and their intercluster proximity is minimal. The clustering of gene expression data makes it possible to identify previously unknown functions of genes that manifest themselves in the expression of a large number of genes. Genes with similar expression patterns (co-expressed genes) can be grouped into clusters, along with those with similar cellular functions. Co-expressed genes in the same cluster tend to be involved in the same cellular processes, and a strong correlation of expression patterns between these genes indicates co-regulation. According to the human protein atlas, HMGB2 is part of cluster 75. The 15 nearest neighbors of HMGB2, based on tissue RNA expression, are presented in the Table 1. The RNA expression clusterization of HMGB2 with bone marrow, cell cycle and mRNA splicing RNA expression data can be observed.

Skin cells, the appendix, the spleen, lymph nodes, tonsils, bone marrow, the thyroid gland, intestinal tissues and testes are all characterized by a high level of HMGB2 expression [59,60,61], while in the cerebral cortex, the cerebellum, liver, pancreas, gallbladder and soft and adipose tissues, its expression is maintained at a low level [58]. It has been established that HMGB2 is highly expressed in undifferentiated myoblasts, which contributes to the regeneration of muscle tissue [62]. Interestingly, HMGB2 is also expressed in all immortalized human and mouse cells, possibly helping to overcome replication limitations [63]. In experiments with mice, *HMGB2* gene knockout has been shown to be non-lethal (unlike *HMGB1* knockout mice which do not reach reproductive age), but males have reduced fertility and impaired spermatogenesis [64,65,66]. *HMGB2* knockout female mice show oocytes which are both decreased in number and are less likely to be fertilized [67]. Moreover, HMGB2 is important for the development and maintenance of ovarian follicles in mice, as *HMGB2* knockout results in ovarian atrophy and fibrosis [61]. It should be noted that in some species, for example, in sharks, a high level of HMGB2 expression persists in all tissues even after birth [68]. HMGB2 is overexpressed in highly proliferative tissues, including various types of cancer, and is downregulated in senescent cells and tissues [65,69,70,71]. In addition, HMGB2 (but not HMGB1) has been shown to suppress the growth of malignant cells in vivo by controlling the gene expression program responsible for hypertrophic cell growth [72].

**Table 1:** Fifteen nearest neighbors of HMGB2 based on tissue RNA expression. Data are available online in The Human Protein Atlas [58].

Neighbour	Description	Correlation *	Cluster
ORC1	Origin recognition complex subunit 1	0.9930	75
HJURP	Holliday junction recognition protein	0.9842	75
SPC24	SPC24 component of NDC80 kinetochore complex	0.9789	75
TFDP1	Transcription factor Dp-1	0.9719	75
ING3	Inhibitor of growth family member 3	0.9684	75
MCM2	Minichromosome maintenance complex component 2	0.9684	75
PCLAF	PCNA clamp-associated factor	0.9667	75
DNAJC9	DnaJ heat shock protein family (Hsp40) member C9	0.9649	67
CDCA5	Cell division cycle-associated protein 5	0.9649	75
ATAD5	ATPase family AAA domain containing 5	0.9614	75
CDC25A	Cell division cycle 25A	0.9596	75
RFWD3	Ring finger and WD repeat domain 3	0.9596	75
E2F2	E2F transcription factor 2	0.9596	75
CDT1	Chromatin licensing and DNA replication factor 1	0.9579	67
CENPN	Centromere protein N	0.9579	75

* Correlation between the selected gene and neighboring gene is calculated as Spearman correlation in PCA space based on the RNA-seq expression data.

## 4. Post-Translational Modifications of HMGB2

Post-translational modifications (PTMs) of HMGB2 are still poorly understood. However, it should be noted that, similar to HMGB1, PTMs determine the localization of HMGB2 in the cell and, therefore, affect its functions. HMGB2 undergoes acetylation, phosphorylation, oxidation, and methylation. The vast majority of PTMs described in the literature, such as the acetylation of lysine amino acid residues at positions K3, K43, K90, K114 and K141, phosphorylation at position S100, and the oxidation of cysteine residues at positions C23, C45 and C106, are same as those in HMGB1 [73]. Cysteine residues at positions 23 and 45 are located near amino acids that influence the DNA-binding properties of all four proteins of the HMGB family [74]. It should be noted that cysteine residues 23 and 45 are conserved in human HMGB1-3 proteins. Additionally, in HMGB4, it was found that cysteine residues are located in slightly different positions: C45, C106, C164 and C178 [75]. Cysteine 106 is conserved in all four proteins of this family [76]. It is C106 oxidation state that affects the localization of these proteins in the cell. Here, we discuss some of the modifications in more detail.

### 4.1. Acetylation

The acetylation of amino acid residues in two regions of nuclear localization (NLS: Nuclear Localization Sequence) leads to the release of HMGB1/2 proteins from the nucleus. It has been shown that in the case of HMGB1, this statement is true only for active secretion, and in the case of the passive secretion of the protein, the acetylation of lysines in this region is not required [17].

**Figure 3:**
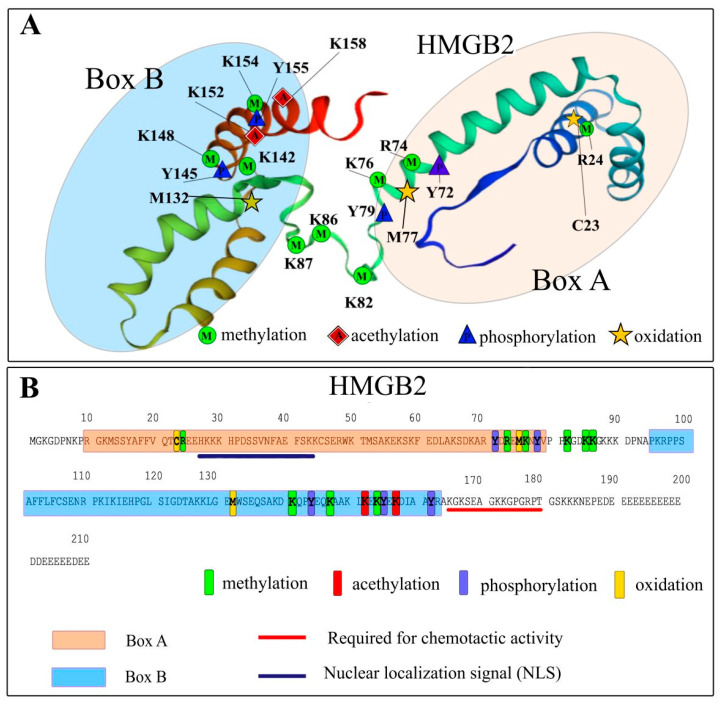
Post-translational modifications (PTMs) of calf thymus HMGB2. Panel (**A**)—schematic representation of potential PTM site location. Panel (**B**)—potential HMGB2 PTM sites are concentrated in the Box B and within the linker region between the two HMGB domains. Figure was prepared using previously published materials [77].

In the work of Bonaldi T. et al. [17], HMGB1 lysins potentially susceptible to acetylation were analyzed. It was shown that K50, K57, K59, K65, K68, K77, K82, K87, K88, K90, K141, K146, K147, K150, K152, K154, K163 and K165 in differentiated cells do not undergo acetylation at all. Data from PTM analysis of calf thymus HMGB2 obtained by MALDI-mass spectrometry are presented in Figure 3. In the case, acetylation did not occur in these sites, with the exception for K152 [77]. However, the functional significance of lysine acetylation at position 152 is still unclear. In some studies, acetylation at position K30 was also noted [78], which is typical for the HMGB domain. When studying the HMGB2 PTMs in the calf thymus, no acetylation was found in the positions K3, K7, K8, K12, K170, K172, K173 and K177 [77], which is consistent with previously reported data on HMGB1 [17].

### 4.2. Oxidation

Along with the acetylation of lysines in nuclear localization regions, an important factor in the intracellular migration of HMGB1 and HMGB2 proteins is oxidation of cysteine residues. As mentioned earlier, cysteine residues at positions C23, C45 and C106 of HMGB1-3 proteins can undergo oxidation (one, two or all three cysteine residues simultaneously), which can lead to the formation of C23–C45 disulfide bridges. Cysteine residues C23 and C45 are conserved in HMGB1-3 proteins [75] and located in DNA-binding domain A near amino acids that affect the DNA-binding properties of HMGB2 and, presumably, of HMGB1 and HMGB3 [74].

It has been shown that in the cell nucleus, HMGB1 is in a completely reduced form (all three cysteines are reduced) [79], which is probably necessary for the protein to perform structural and regulatory functions in the cell. The high degree of structural conservatism of HMGB1 and HMGB2 suggests that HMGB2 is also in a completely reduced form in the cell nucleus. It is known that the translocation of HMGB1 from the nucleus to the cytoplasm is accompanied by partial protein oxidation (C23 and C45 are oxidized and form disulfide bonds with each other, and C106 remains in a reduced state) [20,79]. We have shown that in the calf thymus, for example, C23 is oxidized in both HMGB1 and HMGB2 proteins, while C45 and C106 have a reduced form [77]. The absence of the oxidation of C45 and C106 may affect the ability of these proteins to bend DNA [43], as well as to interact with non-canonical DNA structures [8,11,13,80]. It has recently been shown that cysteine oxidation can also occur in the nucleus, playing an important role in the interaction of these proteins with DNA [81].

In the case of the non-signaling release of both HMGB2 and HMGB1 into intercellular space under the influence of external conditions, partial protein oxidation also occurs. For example, it was shown that the redox status-mediated localization (cellular vs. extracellular) of HMGB1 directly affects its immunity and autophagy functions [79], as well as its ability to interact with a number of receptors involved in immune response signaling and inflammation.

It has been shown that the oxidation of the HMGB domain has a strong effect on its conformation [82]. The oxidation of HMGB1 leads to the formation of a disulfide bond between C23 and C45, which causes a change in the orientation of the Phe38 aromatic ring, which plays an important role in the stabilization of the DNA–protein complex due to partial intercalation between DNA base pairs. A change in the orientation of the Phe38 ring leads to a decrease in the binding affinity of protein-to-platinum adducts on DNA, which determine the antitumor activity of cisplatin family drugs [80,82].

### 4.3. Phosphorylation

**Figure 4:**
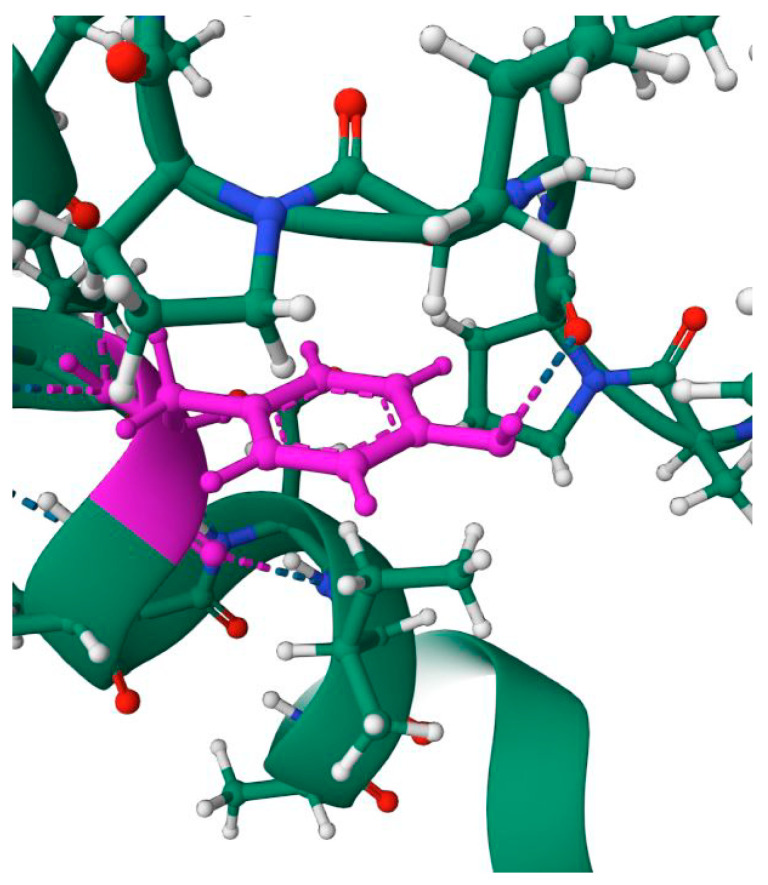
Local structure of Y155 (purple) phosphorylation site. The hydrogen bonding between Y155 and Pro8 is shown. The color scheme is the following: red—oxygen atoms; blue—nitrogen; gray—hydrogen; green—carbon.

The analysis of HMGB2 PTMs showed the presence of several phosphorylation sites at positions Y71, Y78, Y144, Y155 and Y162 [77]. As in the case of HMGB1, the biological role of these modifications has not yet been identified. However, some of them lead to structural consequences that are worth mentioning. For example, the phosphorylation of Y155 disrupts the interaction between Y155 and P8 (Figure 4), which is essential for the stabilization of the overall domain structure. Some of these PTMs are located in the region near the linker between DNA-binding domains. The study of HMGB1 PTMs showed that the phosphorylation (along with acetylation and methylation) of serine residues located near the NLS (S34, S38, S41, S45, S52 and S180) blocks re-entry of the protein into the nucleus, which leads to its accumulation in the cytoplasm [83]. The detected sites in which phosphorylation takes place are located quite far from the NLS regions, and, as in the case of HMGB1, one might assume that the phosphorylation of these amino acid residues should not lead to a change in the protein localization in the cell but may affect the interaction of HMGB2 with DNA and protein partners.

### 4.4. Methylation

**Figure 5:**
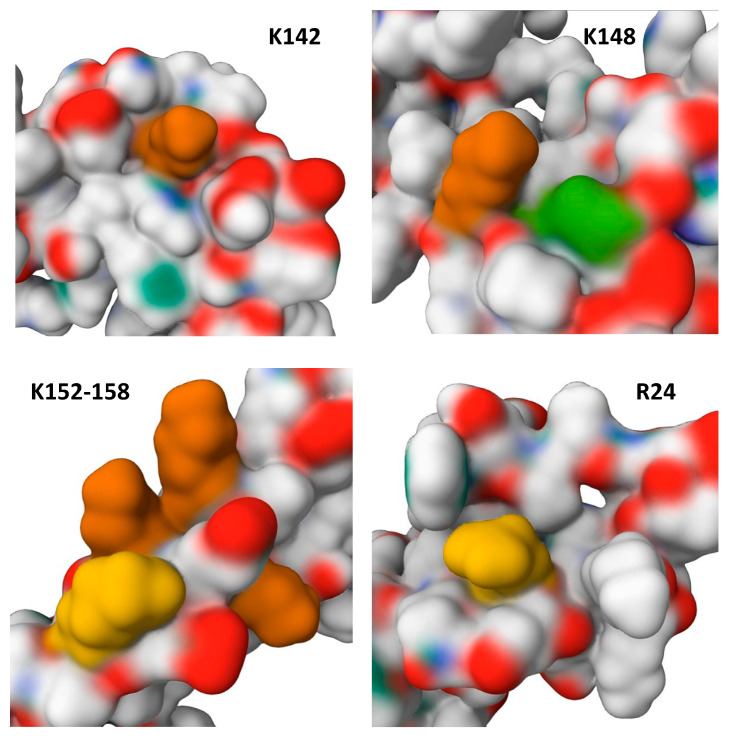
The local surface representation of several methylation sites of HMGB2. Lysines that can be methylated are highlighted with yellowish colors; Y155 residue (also see Figure 4) is marked with green. The images were generated based on the structures of DNA-binding domains of HMGB2 (PDB ID 1J3X and PDB ID 1J3D [84,85].

Recently, the presence of potential methylation sites in HMGB2 at positions R24, R73, K76, K82, K85, K86, K141, K147 and K154 were reported [77]. Most of the identified methylated sites, namely K76, K141, K147, and K154, are highly conserved in HMGB1 and HMGB2. In case of HMGB1, their location corresponds to the positions of “non-acetylated” lysines described in the literature [17]. Reasons for the inability to deacetylate lysines in these sites are probably related to the presence of methylation, which can prevent acetyltransferase from modifying these amino acid residues. Along with acetylation, the charge of these regions also appears to be conservative, which is likely important for the DNA-binding properties of the HMGB domains. The local environment of these methylation sites (Figure 5) demonstrates the very strong structural impact of PTMs, which also explains their conservatism and possible effect on intermolecular interactions of the HMGB domain.

The remaining potential HMGB2 methylation sites that were identified are located predominantly near the linker between two DNA-binding domains [77]. In HMGB2, in contrast to HMGB1, a number of methylation sites (R73, K76, K82, K85 and K86) are observed in the region of the linker region between A and B domains. Considering the importance of acetylation at the K82 position in the HMGB1 linker region for its functional activity, it can be assumed that the accumulation of methylation sites identified in this region in HMGB2 may also be functionally significant.

Thus, most PTMs of calf thymus HMGB1 and HMGB2 reside in areas with high structural conservatism. However, PTMs of HMGB1 are located predominantly in the DNA-binding Box A of the protein and in the linker region between two HMGB domains. PTMs of HMGB2, on the contrary, are concentrated in the Box B and linker region of the protein.

## 5. Biological Functions of HMGB2 in Cell Nucleus

**Figure 6:**
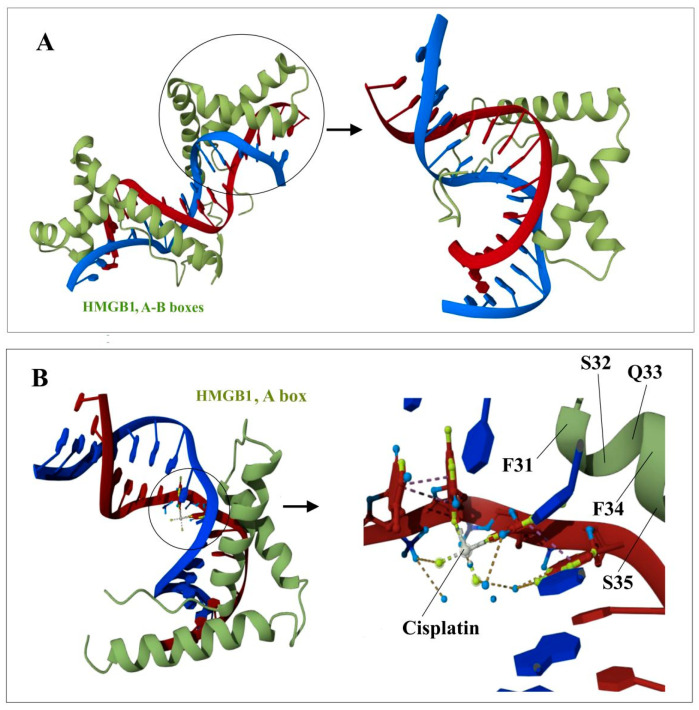
Structure of the complexes between the DNA-binding HMGB-domains and DNA (panel (**A**)); DNA modified by cisplatin (panel (**B**)). The color scheme is the following: red and blue—DNA chains; green—protein; grey—platinum; light blue—oxygen atoms; light green—nitrogen atoms. Images were created using NMR structures, deposited in Protein Data Bank (PDB IDs are 2GZK [153] and 1CKT [74], respectively).

**Figure 7:**
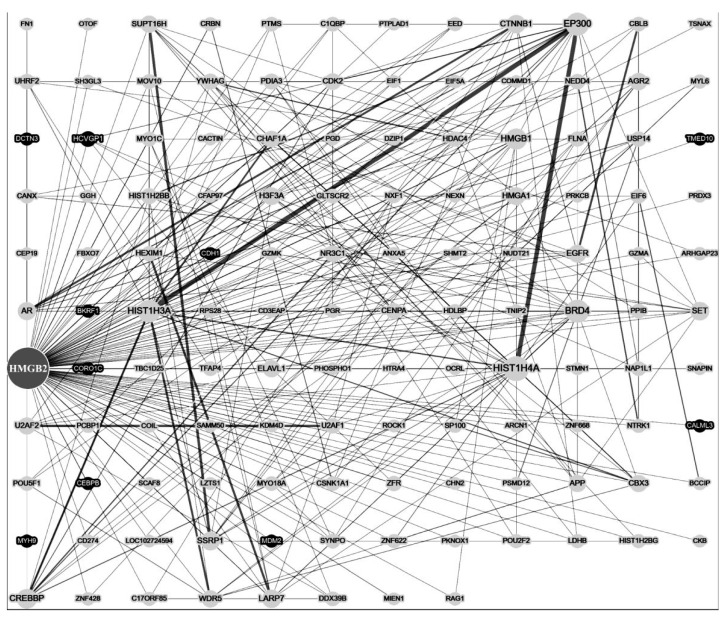
STRING protein–protein interaction network for human HMGB2 protein. The most interconnected component of the networks of interactions of the HMGB2 protein with the protein components of human cells. The network was built using the STRING database [160].

As we noted earlier, the HMGB2 protein is involved in many cellular processes, such as the transcription, replication, and recombination of DNA. At the same time, one of the main functions of this protein is based on its DNA-binding properties—the protein takes part in the remodeling of chromatin and, as a DNA chaperone, in the main DNA-dependent processes (Figure 6). At the same time, an important aspect of HMGB2 functioning is an extensive network of protein–protein interactions (Table 2, Figure 7), which affects the role of this protein not only in the nucleus, but also outside of it. Below, we focus on some functional features of this protein.

### 5.1. Interaction with DNA

The HMGB2 protein (such as HMGB1) binds double-stranded DNA in a non-sequence-specific manner [143,144]. However, both proteins show selectivity and a high affinity for supercoiled plasmid DNA [86,87,88,89], cruciform DNA structures [90,91], DNA modified with cisplatin [96,97,98,99,145] and B-Z cross structures [87,90,91]. It has also been shown that HMGB2 (as well as HMGB1) binds cooperatively to DNA minicircles [146,147].

The interaction of the HMGB domain with double-stranded DNA occurs within its minor groove, while aromatic amino acid residues intercalate between DNA base pairs unwind the duplex and induce a strong bend of the double helix towards the major groove [92,93]. Binding of various HMGB proteins to DNA results in bending of the double helix with angles ranging from 30 to 130°. For single-domain HMGB proteins, typical binding angles vary from 70 to 90° [148,149,150]. It has also been shown that the isolated Box A of HMGB2 is characterized by a rather weak DNA bending ability compared to that of the isolated Box B or recombinant protein containing both DNA-binding domains connected by a linker [14,151].

**Figure 8:**
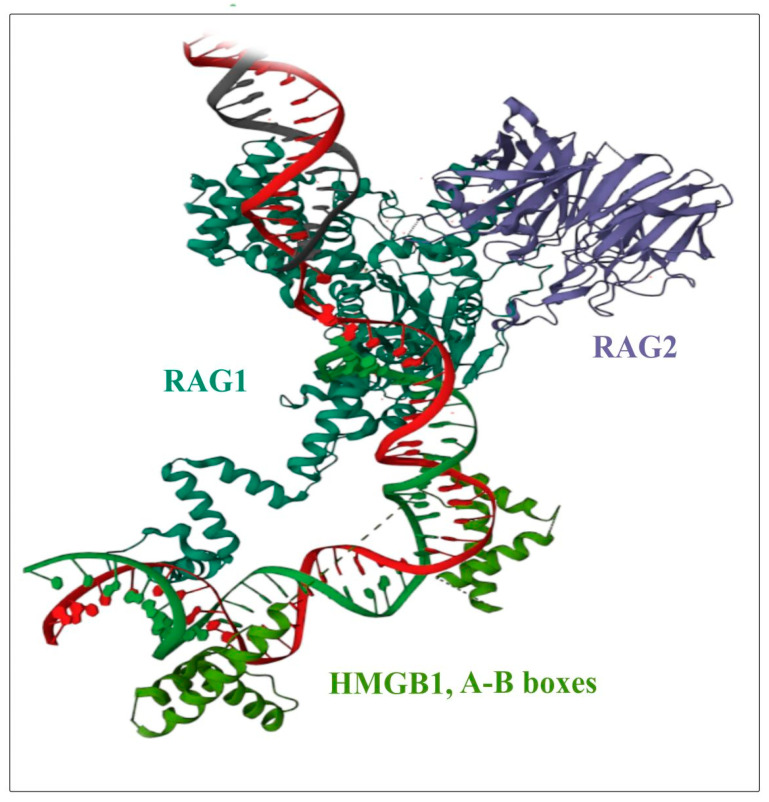
Structure of the complex between HMGB-domain (light green), DNA (green and red chains) and proteins RAG1/2 (green/purple respectively). The image was generated using NMR structure, deposited in Protein Data Bank (PDB ID is 5ZDZ [123]).

**Figure 9:**
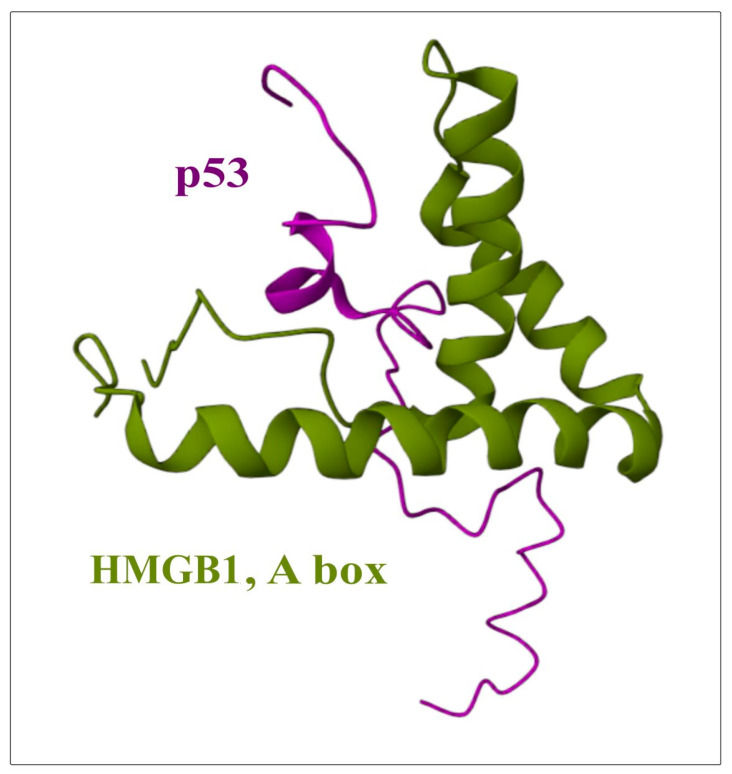
Structure of the complex between HMGB-domain (green) and the transcription factor (oncosuppressor) p53 (purple). The image was generated using NMR structure, deposited in Protein Data Bank (PDB ID is 2LY4) [51]).

Despite the fact that a large amount of experimental data has been accumulated on the interaction of HMGB2 with DNA and other proteins, to date, to the best of our knowledge, there are no published direct structural experiments on the structures of complexes with HMGB2, such as those using NMR or X-ray crystallography. Figure 6, Figure 8 and Figure 9 show models of the interaction of the DNA-binding domains of HMGB1 with DNA, proteins RAG1/2 and with the transcription factor (oncosuppressor) p53. The presented structures were obtained using NMR. The molecules shown in Figure 6, Figure 8 and Figure 9 interact with both HMGB1 and HMGB2 proteins (Table 2).

According to some data, a single B domain of the HMGB2 protein induces a DNA bend of ~99 ± 9°, while two B domains (for example, HMGB1) together bend the DNA molecule only by ~77 ± 7° [94]. Due to the ability of HMGB proteins to bend DNA, these proteins influence the structure and stability of DNA–protein complexes in the immediate vicinity of their binding sites. The induction of DNA bends and recognition of distorted DNA structures are two of the main mechanisms for the functioning of HMGB domain proteins as DNA chaperones [95]. The formation of a DNA bend upon interaction with HMGB1/2 initiates the attachment of the chromatin–remodeling complex, which performs nucleosome sliding until the DNA region becomes accessible to transcription factors. In this regard, HMGB1 and HMGB2 are considered to be proteins that promote the binding of transcription factors [13,106] via the formation of temporary protein/protein contacts with these factors [13,46,47,48,121,135,152]. Figure 6 shows models of the interaction of the DNA-binding domains HMGB1 with DNA [153] modified by cisplatin [74]. The presented structures were obtained using the NMR method.

The aging process of various cell types is also directly related to a change in the structural organization of the genome. In the late 1980s, a hypothesis was put forward, according to which the entire chromatin of a eukaryotic cell is divided into structural and functional domains [70]. According to this hypothesis, the chromatin domain is a loop containing one or more genes, the ends of which are anchored in the nuclear matrix. It has been shown that the HMGB2 protein causes the of loops that represent functional domains along the chromosomes. Individual loops are characterized by independent DNA supercoiling. The chromatin of one domain can undergo conformational transition into an open (transcriptionally active) or closed (inactive) state independent of other domains [154,155]. It is assumed that the mechanism of chromatin loop formation by HMGB2 may be similar to the mechanism of loop formation by CTCF (CCCTC binding factor), an evolutionarily conserved vertebrate transcription factor that binds to various functional elements of the genome and performs various regulatory functions [131,156,157]. The suppression of HMGB2 triggers three key features that are characteristic of a cell undergoing the aging process: reduced amounts of nascent RNA, a shift from facultative to constitutive heterochromatin and the destruction of boundaries of topologically associated domains (TAD) associated with HMGB2. However, unlike HMGB1 [158], the loss of HMGB2 is not sufficient to induce aging. In addition, it turns out that HMGB2 is associated with mitotic chromosomes in the same way as Sox2 is [152]. In the work of Zirkel et al. [70], it was suggested that HMGB2 depletion may lead to disturbances in the structural organization of higher order chromatin. This may be an important step in the subsequent aging program for all cell types, and HMGB2 itself acts as a topological isolation rheostat, the depletion of which reversibly affects global transcriptional competence and heterochromatin organization.

Lee et al. [159] identified a number of amino acid residues of the CIC domain of Capicua-HMG-ETV5 that are essential for the interaction of the HMG domain with DNA. Some of the identified amino acid residues are highly conserved in most of the HMG domains of various proteins, such as R202 and R215. R202 and R215 CIC of the Capicua-HMG-ETV5 domain, corresponding to R97 and R110 B-Box of HMGB1, respectively. R202A, N205A and R215A (R97, S100 and R110 in B Box of HMGB1, respectively) mutants are known to completely lose their binding abilities to target DNA. At the same time, it was also shown that R202Q and R202G substitutions lead to a slight decrease in the efficiency of binding the CIC domain to DNA.

### 5.2. Interaction with Protein Partners

HMGB1 and HMGB2 are similar in their DNA-binding properties. However, their protein partners are significantly different [13,75]. Using various approaches, a number of proteins interacting with HMGB2 were identified (Figure 7, Table 2).

The PPI network (Figure 7) shows possible interaction/colocalization of the HMGB2 protein with many protein components of cells. In this paper, we consider the most significant proteins, for which the interaction with HMGB2 might play important roles in the functioning of chromatin, DNA and the cell as a whole, as well as the possible role of these interactions in the context of certain diseases (Table 2).

First, HMGB2 is able to bind to its paralog, the HMGB1 protein [106,121]. These proteins share a number of common partner proteins, such as nuclear hormone receptors [75]. In the nucleus, HMGB2 (just as HMGB1) interacts with various transcription factors. One example of transcription factors interacting with HMGB2 are members of the POU-domain family [46]. It has been shown that HMGB2 interaction with Oct2 leads to an increase in DNA sequence-specific recognition in vitro, as well as an increase in transcriptional activity in vivo. HMGB2 can also interact with other members of the POU-family—Oct1 and Oct6 [46] and, due to the high conservatism of the POU domain, with other members of this family. Also, the POUh sub-domain through which Oct2 binds HMGB2 is a classical homeodomain which is present in the large class of homeobox proteins, thus extending the range of proteins partners of HMGB2.

Another example of the functional significance of HMGB2 is its role in cartilage development [161]. At multiple stages of cartilage development during embryogenesis, the Wnt/β-catenin signaling pathway is activated [162]. It has been observed that β-catenin and HMGB2’s expression patterns during embryonic development in all areas of the articular cartilage are similar. To substantiate this similarity, functional and physical interactions between β-catenin and HMGB2 were analyzed [69,107,108,109]. It has been shown that the formation of a complex containing HMGB2, β-catenin, lymphoid enhancer-binding factor 1 (Lef1) and probably other components leads to an increase in the expression of genes containing Lef1 binding sites [69,107,108,109]. At the same time, no direct interaction between HMGB2 and β-catenin was revealed, while Lef1 protein interacts with the intracellular domain of Notch family proteins (transmembrane receptor proteins) [163]. It has previously been shown that Notch1 is expressed during the embryonic development of articular cartilage [107] in a spatial-temporal pattern similar to that of HMGB2 [69]. Based on this, the involvement of Notch in the formation of the Lef1-HMGB2 complex was suggested [69,107,108,109].

HMGB2 can interact with steroid hormone receptors (estrogen, androgen and glucocorticoid) and enhance their in vitro binding and transcriptional activity in mammalian cells [137,141]. Apparently, these interactions play an important role in neoplasia since hormonal dependence has been shown for many types of cancer. Among these, the most common are breast, cervical, prostate, and ovarian cancers [75,137,138,139]. Several groups of researchers [65,131,137,164] showed that in vitro both HMGB1 and HMGB2 proteins bind to Hox proteins [131,132,164], steroid hormone receptors [137,140], and RAG1/2 recombinase [48,75,116,119]. The interaction of HMGB1 and HMGB2 proteins with the androgen receptor facilitates the binding of the latter proteins to DNA target sites [137]. Both HMGB proteins can enhance the transcriptional and recombination activity of partner proteins upon transient expression in mammalian cells [116,117,118,119]. Extracellular HMGB2 can bind these receptors, but its affinity to target receptors and its ability to induce inflammation are at a relatively low level, as compared to those of HMGB1 [142]. A model of the interaction of the DNA-binding domain HMGB1 with DNA and proteins RAG1/2 is presented in Figure 8.

Experiments in vivo have shown that a function of this complex is associated with types of cancers that are resistance to chemotherapeutic drugs, in particular, ovarian cancer [75,113,114]. As noted above, a characteristic feature of the HMGB1 and HMGB2 proteins is their ability to recognize and preferentially bind DNA regions with various structural disorders. One example of this form of interaction is the nuclear multiprotein complex which changes the conformation of DNA and defines the sensitivity of cells to chemotherapy. In addition to HMGB2, this complex also includes HMGB1, HSC70, GRP58 disulfide isomerase protein, and GAPD (glyceraldehyde3-phosphate dehydrogenase) [75,113,114,115]. In a study performed on ovarian cancer patients with drug resistance in vivo, an increased expression of HMGB2 and of some metalloproteinases was found. At the same time, genes encoding extracellular matrix proteins and proteins regulating the cell cycle were suppressed in these patients. In a genome-wide search of genes associated with resistance to platinum-based anticancer chemotherapy in ovarian cancer, several genes responding to oxidative stress were found, including those involved in signaling-mediated response via NRF2, P53, and TGF proteins, which are also directly linked to HMGB proteins [75]. A model of the interaction of the DNA-binding domain HMGB1 with the onco-suppressor p53 is presented in Figure 9.

It is known that reversible folding of chromatin fibers into compact structures and the saturation of chromatin regulatory regions with nucleosomes play a key role in the regulation of transcription, replication, and repair [165,166]. At the most fundamental level, the compaction and formation of heterochromatin lead to a decrease in transcriptional activity, replication, and repair, while chromatin loosening, on the contrary, enhances these processes [167,168,169]. HMGB2 has been shown to be one of the components of the SET complex associated with the endoplasmic reticulum. In addition to HMGB2, this complex includes three DNA nucleases (NME1, TREX1 and APEX1), two chromatin modifiers (SET and ANP32A), and tumor suppressor protein pp32 [110,111,112]. This complex is involved in the processes of apoptosis and DNA repair, as well as in the response of cells to oxidative stress [111,170]. It has been established that HMGB2 interacts directly with SET protein, and, in this regard, it has been suggested that HMGB2 can promote the SET-associated nucleosome assembly. Just as HMGB-domain proteins bend DNA around the enhanceosome [171], HMGB2 can bend DNA during the assembly/disassembly of the nucleosome. Since HMGB2 recognizes structural abnormalities in DNA with high efficiency, it has been hypothesized that the binding of HMGB2 to damaged DNA can facilitate the binding of SET and APE1 at DNA damage sites, which subsequently leads to nucleosome unwinding and DNA repair [110].

A number of studies have shown interaction of the HMGB1 protein with the linker histone H1 [35,39,40,41,42,43,44,45,172,173]. Surprisingly, however, no studies on the interaction of HMGB2 with histone H1 could be found. It is known that both HMGB2 and HMGB1 interact with the inter-nucleosomal region of DNA, along the minor groove, and bend the DNA molecule. Taking into account the similarity of the amino acid sequences of these proteins, one can assume that HMGB2 should also directly interact with histone H1. However, in order to confirm this possibility, additional experimental studies are required.

### 5.3. HMGB2 as an Alarmin

As already noted, the HMGB2 can be attributed to the group of alarmins that attract leukocytes and activate dendritic cells, initiating the innate and adaptive immune responses. Alarmins recognize different types of receptors, specifically, HMGB2 and HMGB1 act through the glycosylation end products (RAGE) receptor. Recently, studies have appeared that consider the role of this receptor in osteoporosis [59,136]. RAGE plays a key role in the regulation of bone metabolism under physiological conditions and may contribute to a number of bone-related diseases, including osteoporosis. It is the receptor HMGB-domain proteins bind to trigger intracellular signal transduction. HMGB1 and HMGB2 act on the RAGE receptor in osteoclast progenitors (multinuclear cells that degrade collagen and bone minerals), and the HMGB/RAGE complex modulates cytokine expression and affects osteoclastogenesis in pathological conditions. HMGB1 and HMGB2 proteins are required for the formation of osteoclasts. Treatment with the RANKL protein (TNFSF11, a membrane protein and cytokine of the tumor necrosis factor family) stimulates HMGB1 and HMGB2 proteins to bind to the RANKL-sensitive sequence on TNFα (tumor necrosis factor α) gene promoter and enhances its transcription. Thus, HMGB proteins and TNFα play crucial roles in the regulation of osteoclastogenesis and bone tissue remodeling [59,110,111,115].

Several authors have concluded that HMGB2 is largely involved in processes of inflammation [69,108,174] and cancer development [175,176], as well as in the response of the immune system to these processes [174]. The onset of inflammation can increase the production of ROS, and hence, enhance the inflammatory response. HMGB1 and HMGB2 are highly expressed in immune cells, and in addition, these proteins are associated with hormone-dependent cancers such as ovarian and prostate cancers.

## 6. Effects of HMGB2 Expression Level on Cell Viability

### 6.1. Decreased HMGB2 Expression

The expression level of HMGB2, in contrast to that of HMGB1, is more often associated with cell differentiation, cellular senescence, and the ability of embryonic and adult stem cells to differentiate [109,177,178].

It has been shown that 3T3-L1 preadipocyte cells with a knockdown of HMGB2 via small interfering RNA (siRNA) have impaired differentiation into adipocytes [179]. It is known that β-catenin is degraded during adipocyte differentiation. As mentioned above, β-catenin and HMGB2 are characterized by co-localization and similar expression patterns during cartilage development. The formation of a complex containing HMGB2, β-catenin, Lef1, and probably other components leads to an increase in the expression of genes containing Lef1-binding sites [69,107,108,109]. It is known that β-catenin must be cleaved during adipocyte differentiation. However, HMGB2 knockdown does not suppress β-catenin. This circumstance may be one of the reasons for the absence of adipogenic differentiation in HMGB2-deficient cells [179]. In vivo, the deletion of HMGB2 promotes weight loss, reduces fat deposits, and attenuates adipogenesis in 10-week old mice. As it turns out, the knockout of the *HMGB2* gene leads to a decrease in the expression of PPARγ, C/EBPα, and FABP4 [180], which are supposed to be strongly expressed during the terminal differentiation of adipocytes. It has been established that HMGB2 plays an important role in the regulation of adipogenesis via binding to the C/EBPβ promoter at the stage of mitotic clonal expansion (MCE). Overexpression of HMGB2 promotes adipogenesis and the transformation of fat into skeletal muscle via PDGFRα (platelet growth factor A receptor) [135]. The interaction of HMGB2 with IGF2BP2 plays an important role in skeletal muscle regeneration [62]. In addition, HMGB2 can regulate MSC chondrogenesis [109].

HMGB2 knockdown leads to a decreases C/EBPβ (CCAAT-enhancer-binding protein) expression and inhibition of adipogenesis, as well as to a decrease of the size of mature adipocytes and body weight in vivo [181]. A high-fat diet could restore some fat mass in HMGB2 deficient mice but to a lesser extent than it did in the control group. Thus, HMGB2 is involved in the modulation of the early adipogenesis cascade, but not in the formation of lipids themselves.

In addition to the observed decrease of body weight and the weakening of the adipogenic potential in HMGB2-deficient mice, there is a perturbation within the cardiovascular system. AKT-SERCA2a signaling plays an important role in maintaining cardiac contractility [182,183]. To determine underlying mechanisms, Michio Sato et al. [184] evaluated the potential changes in AKT-SERCA2a signaling in *HMGB2* knockout cardiomyocytes. It was shown that *HMGB2*^−/−^ cardiomyocytes are characterized by a significant decrease of the level of SERCA2a. A decrease of AKT phosphorylation at both Ser473 and Thr308 was also observed in *HMGB2* knockout heart cells. Taken together, these data indicate that loss of HMGB2 inactivates the AKT-SERCA2a cascade. Mitochondrial energy metabolism also plays an important role in maintaining cardiac contractility [185]. It was shown that in the hearts of 12-week-old *HMGB2*^−/−^ mice, there were no changes in expression of genes associated with energy metabolism or encoding components of the mitochondrial complex [184]. This suggests that the loss of HMGB2 impairs cardiac contractility primarily through inactivation of AKT-SERCA2a signaling.

Oxidative stress activates innate and adaptive immune cells, leading to the release of cytokines and inflammation, such as photoreceptor pyroptosis. ROS initiate inflammation in the retina, which leads to abnormal protein aggregation, increased recruitment of immune cells, and the death of RPE (retinal pigment epithelium) cells and photoreceptors [186]. HMGB2 is found in the outer nuclear layer and in the inner and outer segments of retinal photoreceptors. Under oxidative stress associated with cell death, HMGB2 in photoreceptors moves from the nucleus to the cytoplasm, and then to the extracellular space. One study showed that a knockdown of HMGB2 improved photoreceptor degeneration and reduced the expression of pyroptosis-associated proteins, as well as it increased expression of Nrf2 (nuclear erythroid factor 2) and its target – HO-1 [134]. Elevated levels of HMGB2 (possibly through interaction with RAGE) and NLRP3 (a cytosolic protein involved in caspase activation) induce production of illexin via the NF-kB signaling pathway [134]. A knockdown of HMGB2, on the contrary, leads to an increase in the use of the Nrf2/HO-1 signaling pathway and suppression of the NF-kB/NLRP3 pathway. Thus, HMGB2 knockdown suppresses photoreceptor degeneration in mice with light-induced retinal damage, which could probably be exploited for therapeutic purposes.

HMGB2 is specifically expressed in the superficial zone of human articular cartilage, and the aging of this tissue is associated with the loss of HMGB2 expression [69,108]. Additionally, the expression of HMGB2 decreases during the aging of normal primary human embryonic fibroblasts induced by RAS oncogene [187]. RAS-membrane-bound proteins which carry out one of the first stages of signal transduction and, as a rule, regulate cell reproduction, attachment to the extracellular matrix, state of the actin cytoskeleton, malignant transformation, and other processes. HMGB2 expression is reduced or even completely lost in various tumor cell lines with telomerase deficiency achieved via the expression of dominant-negative hTERT mutant. The protein was also absent in diploid human fibroblasts in the end of their replicative capacity and in the colon of aging mice with telomerase *TERC2* knockout [188]. Studies on IMR90 cell culture have shown that HMGB2 expression declines during aging [187]. The age-related loss of HMGB2 expression contributes to the development of osteoarthritis [108], while the level of HMGB1 expression, according to immunostaining, is not affected. In the first phase of cellular senescence, proliferation-promoting genes are silenced by chromatin compaction in heterochromatic regions. At the same time, genes encoding secreted factors, such as cytokines and chemokines, are actively transcribed. Later, the loss of HMGB2 during aging leads to the spread of repressive heterochromatin to these actively transcribed gene loci [187].

Some evidence also suggests that HMGB2 expression is directly related to the presence of adult stem cell populations in some tissues. In articular cartilage, for example, HMGB2 is expressed in mesenchymal stem cells [109,127]. At the same time, as mentioned earlier, HMGB2 expression decreases with the differentiation of chondrocytes [108]. It has been suggested that the age-related loss of HMGB2 in articular cartilage may be responsible for the decline of the cartilage stem cell population [109].

As HMGB2 depletion occurs with ageing, this may be the reason for the decrease in the number of stem cells in cartilage tissue in an adult organism. In addition, it has been shown that the level of HMGB2 expression in the hippocampus is limited by the population of neural stem cells, which is directly associated with proliferation [189].

Expression of HMGB2 also correlates with the number of hematopoietic stem cells and their ability to regenerate [190]. Hematopoietic stem cells are responsible for the lifelong production of blood cells and undergo self-renewal through a process of constant division in order to maintain a pool of stem cells vital for the survival of the organism. Latexin has been shown to be a negative regulator of hematopoietic stem cells in mice [190]. The expression of latexin inversely correlates with the number of hematopoietic stem cells. It has been shown that HMGB2 knockdown leads to an increase in latexin expression at both the transcript and protein levels, which in turn reduces the number of hematopoietic stem cells and their ability to regenerate in vivo [190]. Given the effect of HMGB2 on stem cell pluripotency [47], the suppression or neutralization of HMGB2 appear to be an option for eliminating cancer stem cells and preventing the development of secondary tumors [127].

The maintenance of HMGB2 expression at a basal level is essential for satellite cell proliferation and muscle regeneration [191]. It has been shown that HMGB2 interacts with ribosomal protein kinase S6 beta-1 (S6K1) and regulates its activity during cell proliferation. The inactivation of S6K1 in C2C12 cells leads to a disruption of cell proliferation and differentiation. Thus, HMGB2 is required for the development and regeneration of skeletal muscles, supporting myoblast proliferation through the regulation of S6K1 kinase activity.

It has also been shown that cells with HMGB2 knockdown are more sensitive to radiation, even when they have been exposed to a relatively low dose (0.5 Gy) of ionizing radiation. HMGB2 is required to protect cells from DNA damage and to effectively repair DNA breaks due to exposure to ionizing radiation [192].

HMGB2 has a limited expression pattern, suggesting that it is required only in specific cells and under specific conditions. Some studies have shown that HMGB2-deficient mice are healthy and have a normal lifespan, while HMGB1-deficient mice die before reaching the reproductive age [64]. These data disprove the hypothesis that HMGB2 is required for normal cell cycle progression [193] and suggest that the downregulation of HMGB2 in human fibroblasts derived from the elderly [194] does not cause aging per se.

### 6.2. Increased HMGB2 Expression

An increase in HMGB2 expression is often associated with various pathological processes. HMGB2 has been shown to be an important protein in carcinogenesis. Authors conducting the bioinformatics analysis of 220 differentially expressed genes [195] identified 12 hub genes that play key roles in oncogenesis, particularly in the development aggressive forms of cancer such as pancreatic cancer. *HMGB2* was one the 12 identified hub genes.

Results of in vitro and in vivo studies indicate that the HMGB2 is required for the proliferation of mouse hepatocyte cells [196]. HMGB2 expression is significantly upregulated in gastrointestinal stromal tumors [176,197], hepatocellular carcinoma [175], cervical cancer [197], breast cancer [71], osteosarcoma [198], pancreatic cancer [199], and other types of malignancies. High HMGB2 expression is associated with a poor prognosis for the patient, as it promotes cell proliferation and glycolysis in cancer cells [71,176,198]. It is also known that the suppression of HMGB2 significantly reduces proliferation, invasion, and glycolysis in tumors [176,200]. High levels of HMGB2 correlate with the primary tumor size, invasion capacity, and the stage of tumor development [50,71,175,197]. Recent experiments have shown that malignant cells contain significantly more protein than neighboring normal tissues [197,201,202,203], which was reliably confirmed in the study of the mechanisms of development of non-small-cell lung cancer [201,202,203] and adenocarcinoma of the lung [204]. Additionally, high expression of HMGB2 correlates with metastasis and, in some cases, with a decrease in sensitivity to certain anticancer drugs, such as cisplatin [98], leading to a resistance of cancer cells to chemotherapy and poor prognosis. HMGB2 enhances tumor cell proliferation through the AKT signaling pathway [50,126]. On the one hand, HMGB2 prevents cell apoptosis. Yet, in some types of cancer (e.g., melanoma), HMGB2 promotes cell proliferation and invasion through the interaction with β-catenin. In case of glioblastoma, the protein regulates activities of p53 and p73 [142]. HMGB2 promotes DNA binding of p53 and p73 proteins [50,142], activates p53, and enhances Wnt/β-catenin signaling. HMGB2 may be also involved in regulation of p53 and MMP-2/TIMP2, leading to a resistance to TMZ chemotherapy [50]. Increased expression of HMGB2 protein affects cell migration and invasion. The influence of HMGB2 on the stability of p53 protein in HeLa cells has been established [205]. Co-expression of HMGB2 and HPV E6 has been shown to prevent HPV E6-mediated ubiquitination and the degradation of p53, which leads to the accumulation of p53 in the cell. At the same time, HeLa cells transfected with HMGB2 are characterized by a decreased proliferation and cell cycle arrest, mainly in the G1 phase. Taken together, these results suggest that HMGB2 can stabilize p53 by interfering with E6/E6AP-mediated p53 degradation in HPV-positive HeLa cells [205]. It is important to note that the HMGB2-dependent increase in p53 stability is specific to HPV-positive HeLa cells, as HCT116 and MCF7 cell lines do not demonstrate this feature [205].

**Figure 10:**
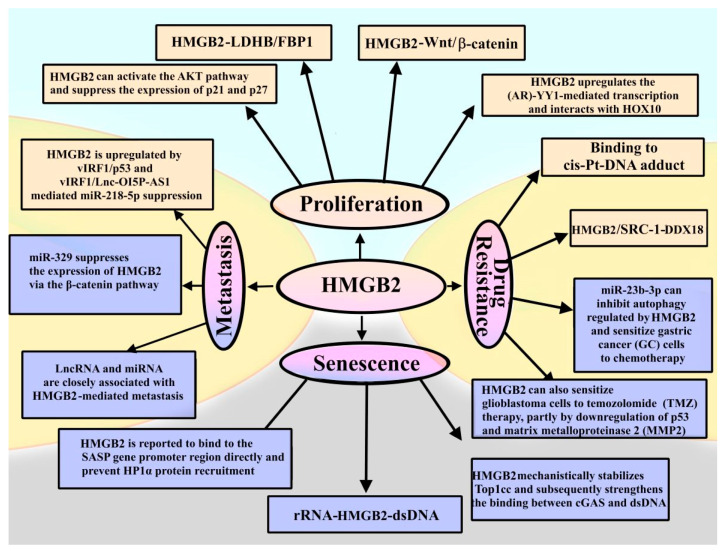
The role of HMGB2 in the development of the diseases. Roles of HMGB2 in tumor proliferation and metastasis: the protein can promote cancer cell proliferation by interacting with HOX10 [132]. It has been shown that HMGB2 mediates the Warburg effect by transcriptional regulation of the activity of glycolytic enzymes lactate dehydrogenase and fructose bisphosphatase 1 (FBP1) [71]. This provides the necessary energy for the proliferation of cancer cells. Moreover, HMGB2 can stimulate p53 or the Wnt/β-catenin pathway [175], activate the AKT signaling pathway through AKT phosphorylation and downregulate p21 and p27 expression in cervical cancer [50]. HMGB2 also plays an important role in the invasion and migration of tumor cells. Non-coding RNAs, including long non-coding RNAs (LncRNA) and microRNAs (miRNAs), are closely associated with HMGB2-mediated metastasis [105]. In Kaposi’s sarcoma (KS) induced by human immunodeficiency virus (HIV), HMGB2 is upregulated by miR-218-5p, mediated by vIRF1/p53 and vIRF1/Lnc-OI5P-AS1 [125]. miR-329 can downregulate HMGB2 expression within the β-catenin signaling pathway in melanoma, ultimately suppressing metastasis [133]. In aging, HMGB2 binds to the promoter region of the SASP gene and prevents the recruitment of heterochromatin protein 1α (HP1α), subsequently regulating the aging processes [123]. HMGB2 mechanically stabilizes the TOP1cc complex and subsequently enhances the binding between cGAS and dsDNA [101]. The HMGB2-dsDNA pathway is also reported to be stimulated by rRNA biogenesis. Inhibition of rRNA biogenesis can stabilize p53 and suppress HMGB2 expression in a p53-dependent manner [124]. Role of HMGB2 in drug resistance: HMGB2 can bind to platinum adducts on DNA and activate DNA repair system, leading to chemoresistance to cisplatin [100]. HMGB2 can bind to the SRC-1 steroid receptor coactivator, and then this complex binds to the DEAD-box helicase 18 promoter (DDX18), resulting in tamoxifen resistance [140]. It is known that miR-23b-3p can inhibit HMGB2-regulated autophagy and increase the sensitivity of gastric cancer (GC) cells to chemotherapy [206]. In addition, HMGB1/2 can increase the sensitivity of glioblastoma cells to temozolomide (TMZ) therapy by suppressing p53 and matrix metalloproteinase 2 (MMP2) [122]. HMGB2 downregulation is marked in yellow boxes, and upregulation is marked in blue boxes.

Figure 10 summarizes roles of HMGB2 in the proliferation of cancer cells, metastasis, drug resistance and senescence.

Summarizing the presented data (Figure 10), one can note that the roles of HMGB2 in disease development and the mechanisms of its disease-related functioning are largely unknown, and more thorough studies are required. Despite many unanswered questions, HMGB1 and HMGB2 proteins can be used as biomarkers of diseases, as well as therapeutic targets for their treatment.

## 7. Conclusive Remarks

In this review, we attempted to summarize currently available data on the structure and functional properties of the non-histone chromatin protein HMGB2. Undoubtedly, HMGB2 plays an important role in the functioning of eukaryotic cells. For many years, it was believed that HMGB1 and HMGB2 proteins perform similar functions in the cell’s nucleus. The main emphasis was placed on their high structural homology and ability to bend DNA at the site of interaction. However, experimental data that emerged within the last decade revealed more complex picture. Despite the high conservatism of both the amino acid sequence and the structural organization, these proteins are characterized by different expression patterns, tissue specificity, modification status, different patterns of DNA binding and a different protein partners. All these differences between HMGB2 and HMGB1 indicate that, along with the main functions common to all HMGB-proteins, there are a number of unique functional features of HMGB2 and HMGB1 in the cell.

Changes in the expression level of HMGB2, both decreased and increased, are often associated with various pathological processes. In this regard, in the future, HMGB2 can be considered as a biomarker of the early stages of cancer, which helps to assess severity of the disease, predict the effectiveness of treatment and patient survival. It is also known that HMGB2 protein can act as a therapeutic target to prevent bone loss and skeletal fragility caused by aging and inflammation. In addition, it can be considered as a biomarker for the progression and severity of bone disease, which can be used for the diagnosis and follow-up of patients suffering from osteoporosis. Additionally, there is a possibility that in the future, these features of the protein can also be exploited for the diagnosis and treatment of patients with COVID-19, which is associated with disorders in the lung tissue. The development of therapeutic approaches for the use of HMGB2 as a biomarker, and their introduction into medical practice is a promising research avenue. However, there are still many fundamental and applied issues related to the mechanisms of the influence of HMGB2 on the course of various cellular processes in normal and pathological conditions, which today require further a careful and more detailed study of the mechanisms of functioning of proteins of this group in the cell nucleus and beyond.

## Figures and Tables

**Table 2:** Some molecular partners of the non-histone chromosomal protein HMGB2.

Partners		Functions
Nucleic Acids		
**DNA**	In the nucleus, HMGB2 acts as a DNA chaperone; it interacts within the DNA minor groove and bends DNA towards the major one. It shows high affinity and selectivity when binding to supercoiled plasmid DNA [86,87,88,89] (PDB code 2GZK, Figure 6A). Recognizes and preferentially binds to DNA regions with various structural damage: cruciform structures, B-Z cross structures, DNA minicircles, etc. [87,90,91]. Interacts with DNA regions modified by antitumor drugs, including cis-platinum (PDB code 1ckt, Figure 6B). When cell undergoes the aging process, HMGB2 causes the formation of “loops” along chromosomes via the same mechanism as that of the transcription factor CTCF (CCCTC binding factor).	Modulates DNA-dependent processes [8,13,92,93,94,95].
		Binds to platinum adducts in genomic DNA and activate the repair system under the action of cisplatin [96,97,98,99,100]
		Changes in the structural organization of the genome [70,101].
**RNA**	HMGB boxes were detected in proteomic screens aimed at the comprehensive identification of RNA-binding domains in human cells [13,75].	Plays an important regulatory role in the cell [102,103,104,105].
**Transcription factors**		
	HMGB2 facilitates binding of transcription factors with DNA.	Formation of transient protein/protein contacts between transcription factors and the HMGB2 protein [42,46,47,48,106].
Oct2, Oct1 and Oct6	The formation of contacts between the HMGB domain of the protein and the POUh subdomain of Oct2 transcription factors. The interaction of HMGB2 with Oct1 and Oct6 was also demonstrated [46].	An increase in DNA sequence-specific recognition by Oct2 proteins in vitro and an increase in its transcriptional activity in vivo [46].
Oct4	It was shown that post-translational modifications of Oct4 directly affect the binding of the protein to HMGB2.	The existence of the Oct4-Akt-HMGB2 regulatory loop [47,48].
Lef1	Notch1 is expressed during embryonic development of articular cartilage in a spatio-temporal pattern similar to that of HMGB2 [107], indicating the involvement of Notch in the formation of the Lef1-HMGB2 complex. The formation of a complex containing HMGB2, β-catenin, lymphoid enhancer-binding factor 1 (Lef1), and, probably, other components [69,108,109]. HMGB2 interacts with RUNX2 and Lef1 at the proximal *Runx2* promoter containing the TCF/LEF motif.	An increase of the expression of genes containing Lef1 binding sites [69,107,108,109].
		HMGB2 can bind to Lef1, as well as to RUNX2, repressing the activity of the *Runx2* promoter [109]
**Other protein complexes**		
Complex SET	HMGB2 is one of the components of the SET complex, which is associated with the endoplasmic reticulum. In addition to HMGB2, this complex includes three DNA nucleases (NME1, TREX1, and APEX1), two chromatin modifiers (SET and ANP32A) and the tumor suppressor protein pp32. It has been established that HMGB2 interacts directly with the SET protein.	This complex is involved in the processes of apoptosis and DNA repair, as well as in the response of cells to oxidative stress. HMGB2 can promote SET-associated nucleosome assembly [110,111,112]
**Nuclear complex**	It has been shown in vivo that HMGB2 forms a multiprotein complex with HSC70, GRP58, and GAPD.	Influence on resistance to chemotherapeutic drugs in cancer patients, in particular, in ovarian cancer [75,113,114,115]. Altering DNA conformation.
	The nuclear complex of HMGB1, HMGB2, HSC70, GRP58, and GAPD alters DNA conformation [75,113,114,115].	
HMGB1	Interacts with its paralog, the non-histone protein HMGB1 [75].	Functions are unknown.
**RAG1/2 recombinase**	Interaction with RAG1/2 (PDB code 5ZDZ, Figure 8).	Enhancement of transcriptional and recombination activities of partner proteins during transient transfection into mammalian cells [75,116,117,118,119].
**TNF and RANKL(TNFSF11)**	HMGB2 and HMGB1 are required for the formation of osteoclasts. Interaction with the RANKL protein stimulates the HMGB1 and HMGB2 proteins to bind to the RANKL-sensitive sequence and enhances TNF transcription.	HMGB1/2 and TNF play a critical role in the regulation of osteoclastogenesis and bone remodeling [59,110,111,115].
**p53**, p73 and p21	HMGB2 activates p53 or enhances Wnt/β-catenin signaling. HMGB2 may be involved in the regulation of p53 and MMP-2/TIMP2, leading to resistance to TMZ chemotherapy (PDB code 2LY4 Figure 9).	Promotes the binding of these proteins to DNA [49,51,120,121,122,123,124,125].
AKT signaling pathway	HMGB2 activates the AKT signaling pathway.	Proliferation of cervical carcinoma [50,126].
HP1α	HMGB2 binds to the promoter region of the SASP gene.	Preventing recruitment of the HP1α protein [123,127].
MIEN1 and NOP53	The interaction of HMGB2 with proteins responsible for the survival of patients with ovarian cancer was shown on the SKOV-3 and PEO1 cell lines.	Suppression of HMGB2 leads to an increased sensitivity to anticancer drugs [70,104,128,129]. HMGB2 is regulator of cell migration and invasion and apoptosis [130].
CTCF proteins	Influence on expression of genes found in topologically associated domains (TADs).	HMGB2 modulate the global chromatin structure and and prevent clustering of CTCF proteins [70,131].
TBP	Interaction with TATA-binding protein.	Enhances the ability of this protein to interact with DNA [131].
Hox, Rep78 and Rep68 of adeno-associated virus	HMGB2 interacts with these proteins.	The increase in the binding of these proteins to DNA facilitates the formation of nucleoprotein complexes [131,132].
β-catenin	β-catenin and HMGB2 are characterized by colocalization and a similar change in expression levels.	The influence on embryonic development in all areas of the articular cartilage [133].
EBNA1	EBNA1 (encoded by Epstein–Barr virus) binds to cellular chromatin during interphase and mitosis and interacts with HMGB2.	Influence on chromatin during interphase and mitosis [131].
NLRP3	Interacts with a cytosolic protein NLRP3 that is involved in caspase activation.	Induces the production of illexi-pallierNF-kB dependent manner [134].
PDGFRα	Interaction with PDGFRα (platelet growth factor A receptor).	Overexpression of HMGB2 promotes adipogenesis and conversion of fat to skeletal muscle [135].
IGF2BP2	The interaction of HMGB2 with IGF2BP2.	Plays an important role in skeletal muscle regeneration [62].
**Alarmin**		
**RAGE**	HMGB2 belongs to alarmins that recognize different types of receptors, including the glycosylation end products receptor [59].	Recentl studies considered a role of this receptor in osteoporosis [59].
	HMGB domain proteins bind with RAGE during intracellular signal transmission. HMGB1 and HMGB2 act on the RAGE receptor in osteoclast progenitors (multinuclear cells that degrade collagen and bone minerals) [59,136].	HMGB/RAGE complex modulates cytokine expression and affects osteoclastogenesis in pathological conditions [59,136].
**Hormone receptors**		
**Steroid hormone receptors**	HMGB2 can interact with estrogen, androgen and glucocorticoid and enhance their in vitro binding and transcriptional activity in mammalian cells [19,137,138,139].	Plays an important role in the development of cancerous tumors, and steroid hormone signaling is important for normal spermatogenesis [19,137,138,139,140,141].
	Extracellular HMGB2 can bind to these receptors and has an affinity for target receptors [142].	Ability of HMGB2 to induce inflammation is relatively lower compared to that of HMGB1 [142].

Bold items in the left column indicate common molecular partners for the non-histone chromosomal proteins, HMGB1 and HMGB2.

## Data Availability

The data presented in this study are available in the article.
